# Cohort profile: Children in Need Census (CIN) records of children referred for social care support in England

**DOI:** 10.1136/bmjopen-2018-023771

**Published:** 2019-02-22

**Authors:** Emily H Emmott, Matthew A Jay, Jenny Woodman

**Affiliations:** 1 Department of Anthropology, University College London, London, UK; 2 UCL Legal Epidemiology Group, UCL Great Ormond Street Institute of Child Health, University College London, London, UK; 3 Department of Anaesthesia and Pain Medicine, Great Ormond Street Hospital for Children NHS Foundation Trust, London, UK; 4 Thomas Coram Research Unit, Department of Social Science, UCL Institute of Education, University College London, London, UK

**Keywords:** child protection, social medicine

## Abstract

**Purpose:**

The Children in Need Census (CIN) is a case-based administrative dataset on children referred to social care services in England. CIN includes information on the ‘needs’ of children, and whether they received social care support. Local and national government bodies in England currently use CIN for evaluation purposes. Data are accessible to researchers under certain conditions, allowing researchers to investigate the health implications of adverse childhood experiences. However, CIN suffers from lack of metadata, meaning it can be challenging for researchers to process and interpret data, particularly if researchers are unfamiliar with the English children’s social care system. To address this issue, we provide the background to CIN and describe the available data from 2008 to 2016.

**Participants:**

CIN is derived from case records held by English local authorities on all children referred to children’s social care for a ‘needs assessment’, regardless of whether they are eventually assessed as ‘in need of social care support’. Local authorities submit these case records to the UK Department for Education for collation. CIN holds information on an estimated 2.76 million children from October 2008 to March 2016. Since 2013/2014, just under 900 000 children have been recorded in the CIN annually, equivalent to around 8% of children in England (annual prevalence). Approximately, 650 000 children enter or renter the dataset each year, equivalent to 5% of children in England (annual incidence).

**Data summary:**

Of the estimated 2.76 million children in the data, 50% are male and 47% female. 45% are referred to children’s social care services due to abuse or neglect. 10.7% of children in CIN went onto a child protection plan, meaning they were judged to be (at risk of) suffering significant harm.

**Future plans:**

CIN data collection is annual and ongoing. Data from the most recent census period typically become available for researchers in the following Spring.

**Strengths and limitations of the Children in Need Census (CIN).**

- The Children in Need Census (CIN) is the largest data source of potentially vulnerable children in England. An estimated 2.76 million children are in the dataset between 2008 and 2016.
- CIN contains identifiers which facilitates linkage with other datasets, including the school census and children looked after census. In addition to some disabilities data in CIN, there is an opportunity to link to other health records such as Hospital Episode Statistics.
- CIN data structure is complex and may require substantial cleaning.
- Not all children are uniquely identifiable at a national level, and some children may have multiple IDs.
- Information recording and assessment practice vary between census years and local authorities, meaning researchers must be cautious around comparisons over time and between areas.

## Introduction

The Children in Need Census (CIN) is an administrative dataset of children identified as being exposed to, or at risk of being exposed to, various adverse childhood experiences which require social care intervention. Adverse childhood experiences are associated with lifelong negative implications for health,^1^ but prospective health research on vulnerable groups of children is challenging: family characteristics associated with increased risk of vulnerabilities such as abuse and neglect overlap with difficulties in participant recruitment and retention.^2 3^ Studies involving children at risk of harm often rely on small sample sizes^4^ or retrospective reports in adulthood.^1^ Using existing data from services that support vulnerable children is one way of overcoming these challenges. CIN provides such opportunities, being composed of longitudinal case records of potentially vulnerable children who have been referred to children’s social care in England.

The name of the dataset (CIN) refers to a legally defined group of children in the UK (Children Act 1989, s 17(10)), who require additional support from local authorities (LAs) (local government bodies) to maintain or achieve a ‘reasonable standard of health or development’ or who are disabled.^5^ Under this definition, CIN includes (but are not limited to) young carers looking after family members with health problems, disabled children, children at risk of maltreatment and children in care (ie, children looked after by the state). Given its broad legal definition, the criterion of whether a child is ‘in need’ requires discretion, and is open to professional interpretation/varying thresholds. In practice, ‘children in need’ are children judged to require social care support (ie, social services/child welfare support).

In England, children’s social care is provided locally by children’s services across 152 LAs. These LAs have statutory duties to safeguard and promote the welfare of children in their area, primarily through (1) assessing the needs of children referred to them for support, (2) establishing whether they are a ‘child in need’ and (3) providing appropriate social care support to CIN up to their 18th birthday (or up to their 25th for care leavers). Cross-sectionally, just over 3% of children in England (around 390 000 children) are identified as ‘in need’ in England and are receiving some form of social care support from LAs at any one point.^6^

As part of their case management process, all LAs in England record information on children who are referred to children’s services for social care support, and keep track of the children whom they assess as ‘in need’ and go on to support. The CIN census is derived from these case records, annually submitted to the Department for Education (central government body overseeing children’s social care in England) for collation at a national level. With full coverage across England, CIN offers an opportunity to understand the characteristics of children who are referred to and are supported by children’s services, their pathways through children’s social care and, by linking to other datasets, their associations with children’s health-related outcomes.

As an administrative dataset not primarily designed for research purposes, the data providers offer little metadata at present. Comprehending, processing and interpreting CIN can, therefore, be a challenge for researchers, particularly for those without specialist knowledge of the English children’s social care system. To address this issue, we provide an introduction and overview of CIN.

## Cohort description

### Background to the CIN census

CIN is a national, case-level dataset on children who are referred to LA children’s services for social care support in England, with information on their pathways through the local social care system. The latest form of the CIN census was introduced in October 2008,^7^ and LAs annually submit their children’s service case records to the Department for Education.^8^ CIN is composed of case information on all children who are referred to LA children’s services for an assessment of their needs (needs assessment), with data recorded around key service decisions and events for as long as the case remains ‘open’ following a referral. Note, referrals for a needs assessment must first be ‘accepted’ by LAs for it to be recorded within CIN. The criteria of acceptance can vary between LAs due to differences in service structures and the ‘thresholds’ of when a child is judged to be potentially ‘in need’. Cases remain open where LA children’s services are providing or planning to provide support to the child, including financial, practical and informational support. It also includes cases where no support is provided, but there are plans to review and assess the child’s needs at a later date.^8^

CIN covers a wide range of children in need including children in care (n=72 670 on 31 March 2017; 18.7% of children identified as in need) and children on child protection plans (children receiving statutory services for suspected maltreatment; n=51 080 on 31 March 2017; 13.1% of children identified as in need).^6 9^ In addition to children in need identified and supported by LAs, CIN also includes (1) children whose referrals are accepted by children’s services for needs assessments but go onto being assessed as ‘not in need’, (2) children who are not yet born following a prebirth referral for a needs assessment and (3) young people who are aged 18 or over but continue to receive care, accommodation and/or support from children’s services.

Not all children known to children’s services are included in CIN: Disabled children on the LA Disabled Children’s Registers are only included if they are receiving services from LAs, regardless of the fact that they are ‘children in need’ as legally defined.^8^ It also does not include children who are receiving support from LAs through their ‘early help’ services, which are preventative/early intervention services targeting children who are not currently ‘in need’ but are at risk of becoming in need in future.

**Figure 1:**
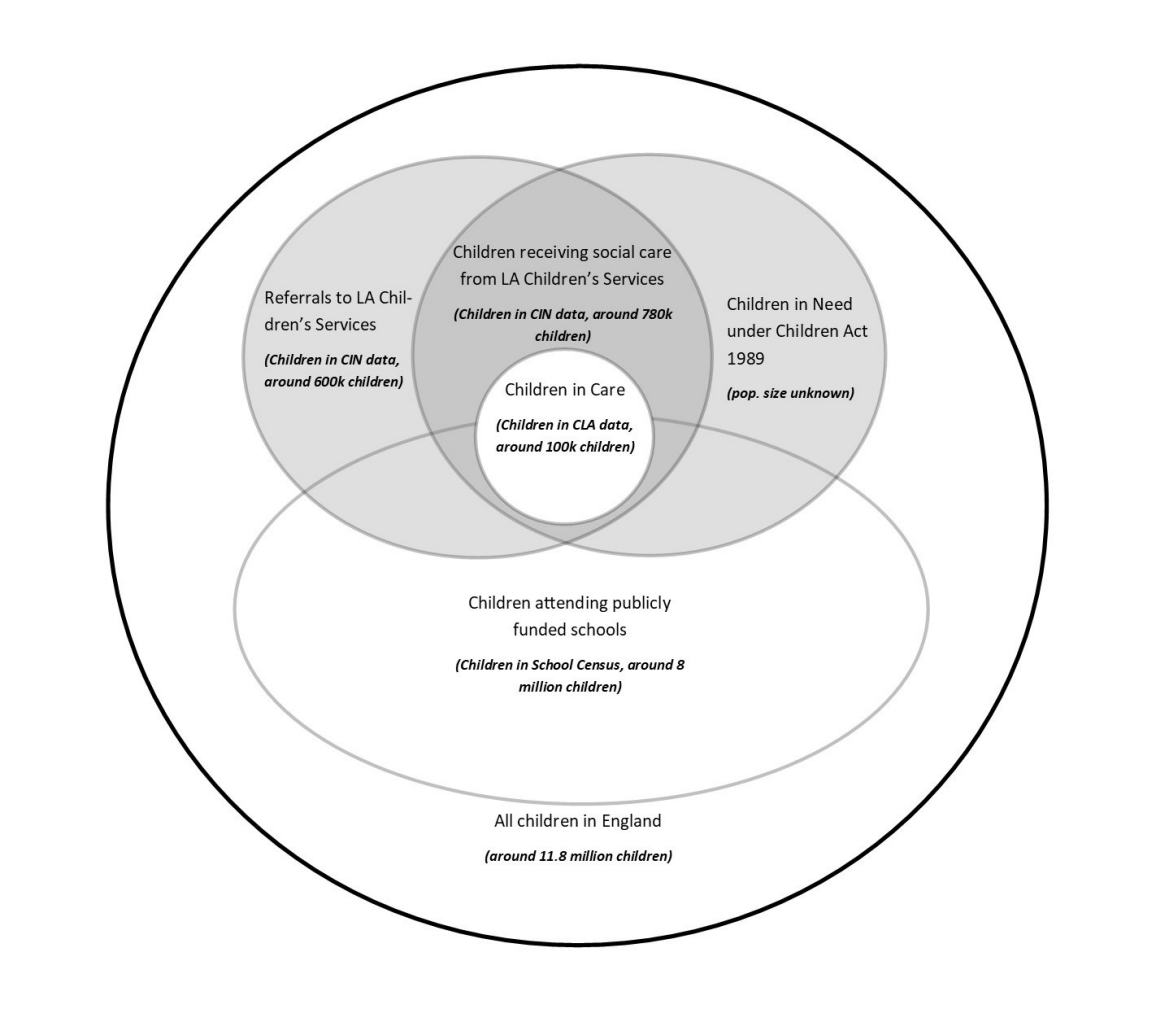
Overlap between the CIN population (as legally defined) and the Department of Education’s National Pupil Datasets (CIN, CLA and School Census). Size and overlaps are not to scale. Child population sizes are estimates for a 12-month period between April 2015 and March 2016. CLA, Children Looked After return; LA, local authority.

CIN is part of the wider National Pupil Database (NPD), which is a collection of different administrative datasets held by the Department for Education on children and young people in England, including the school census and children looked after return (CLA).^10 11^ Children in CIN who attend publicly funded schools can be linked to the school census by their Pupil Matching Reference ID (PMR). Children who are looked after by the state are by definition CIN, and therefore appear in both the CLA and CIN datasets, linked by their LA child ID.^11 12^ The population overlaps in these datasets are represented in figure 1.

According to the CIN data collection guidance for LAs, the purpose of CIN is to track and analyse the journeys of children through children’s services in order to evaluate local and national performance.^8^ CIN largely holds information on service decisions and events, particularly around dates and reasons for action. Since 2012/2013, data collection has expanded to include more information about child protection and referrals. The Department for Education states that linkage to other datasets with information on child outcomes, such as the School Census and CLA, will allow evaluation of the effectiveness of policies and services over time.^8^

### Data collection process

CIN data are extracted from LA children’s services case records, primarily recorded for the purposes of case management. Each year, LA children’s services extract information requested by the Department for Education on all cases open at any point during the census year (1 April to 31 March). LAs are given data specifications drafted by the Department for Education in advance of the census year commencing, and are expected to collect the specified information throughout the year.^13^

LAs submit their extracted CIN data through the Department for Education’s online data collection portal COLLECT, which includes automatic validation checks at the time of submission.^13^ For example, children’s demographic characteristics and referral dates cannot be missing, and queries are automatically raised if the child or young person is aged 25 years or over at the date of referral. Errors must be amended and/or reviewed by LAs before submission. Once submitted and accepted by the Department for Education, LAs cannot amend their submitted data. LAs are however able to retrospectively update their own case records after submission, which may be submitted in the following census years if the case remains open. After submission, aggregate information is visually checked by the Department for Education. If aggregate information seems unusual, the Department for Education contacts the LA to discuss possible issues, and may request the LA to correct and resubmit the source data. The Department for Education does not amend the errors themselves.

The Department for Education does not specify how the data should be collected and extracted, or if and how data should be validated. Based on our experience, LAs collect their CIN data using procured case management systems, where the allocated case worker (ie, social worker) enters details about each case. Several case management systems for children’s services exist on the market, meaning collection methods and internal validation processes likely vary between LAs.

Note, in 2010/2011, the Department for Education coordinated a review of CIN coinciding with the publication of the Munro Review of Child Protection Interim Report.^14^ Archived documents suggest this led to some improvements in the usability of the data submission system and data quality.^14^ This also coincides with a notable increase in the number of children in CIN from the following census year (table 1). The exact cause of this jump in number cannot be discerned from publicly available information.

**Table 1:** The number of LAs, estimated children and episodes in CIN

Census year	2008/2009*	2009/2010	2010/2011	2011/2012	2012/2013	2013/2014	2014/2015	2015/2016	2008–2016
	N	N	N	N	N	N	N	N	N
LA†	148	151	150	150	152	152	152	152	154
Pupil matching references (PMRs) (cleaned)‡	387 413	584 456	501 136	599 425	588 602	618 498	599 898	571 429	1 761 225
LA child ID (derived)§	401 872	595 976	509 088	588 943	818 472	896 471	892 747	885 023	2 761 155
Episodes¶	419 661	714 207	588 943	939 095	941 581	1 021 432	1 018 474	1 000 572	4 668 623

Based on authors’ copy of CIN holding complete episode information between 2008 and 2016. Received from the Department for Education, November 2017.

*Data collection in 2008 began in October rather than April 2008. While retrospective cases were also submitted by many LAs, often going back to or before 1 March 2008, the number of episodes is significantly fewer for this reason.

†In 2009/2010, Bedfordshire Children’s Services (LA ID 820) split into Bedford (LA ID 820) and Central Bedfordshire (LA ID 822), and Cheshire Children’s Services (LA ID 875) split into Cheshire East (LA ID 895) and Cheshire West and Chester (LA ID 896).

‡For children with PMRs in the dataset, PMRs are occasionally missing in some records (particularly during ages when they are older or younger than school age). Different PMRs are occasionally allocated for one child. The displayed figures are for the cleaned PMR variable, after addressing these issues where possible. Further information on the cleaning method is outlined in the online supplementary information.

§LA child IDs do not uniquely identify children, as multiple LAs may use the same ID. Children also acquire new LA child IDs when they move LAs and when they are adopted—meaning we are unable to attribute all records to the same child over time. The displayed figures are for the derived LA child ID variable, combining the original LA child ID and the LA code.

¶The displayed figures include some duplicate cases due to reporting errors, for example, when new cases are opened when children have an existing open case.

CIN, children in need; LA, local authority.

10.1136/bmjopen-2018-023771.supp1 Supplementary data

### Public involvement

CIN is derived from LA children’s service case records, meaning direct engagement with participants around data collection and processing is challenging. As primary data controllers, LAs are required to display and disseminate clear and accessible privacy notices outlining what data is being collected/processed and why, including the lawful basis of data collection and processing (eg, public task, legitimate interest and legal obligation).^8^ Changes to the CIN data collection by the Department for Education require a business-case submission and approval from the Star Chamber Scrutiny Board, which is composed of school and LA representatives.^15^ The top-line findings from CIN is published annually by the Department for Education and is publicly available.^6^

### Data structure

The CIN is an ‘episode’ based dataset, where each episode is an open case following a referral to children’s services. Episodes (ie, cases) are nested within children identified through a national identifier (PMR) or the LA identifier (LA child ID). As such, the dataset is in long format meaning that an individual child can have more than one row of data in the file supplied to researchers. Between October 2008 and March 2016 (based on our CIN data extract), CIN consists of around 4.7 million episodes, attributed to an estimated 2.7 million children across 152 LAs (table 1). Since 2013/2014, just under 900 000 children have been recorded in the CIN annually, equivalent to around 8% of children in England (annual prevalence), and approximately 650 000 children enter or renter the dataset each year, equivalent to 5% of children in England (annual incidence).

Each year, LAs report all open cases over a 12-month period. This means that the same case can be recorded multiple times across a number of years, resulting in duplicate episode entries. New information may also be added to existing cases each year, such as (1) amendments to original records, (2) child protection plan information and (3) case closure information. The most recent record of an episode is most likely to be correct/complete, excluding child protection plan details. As children can have multiple child protection plans within a single CIN episode, child protection plan information must be identified across the recurring records of each episode.

As with all administrative datasets, researchers will need to carry out data cleaning before the data are in a ‘research ready’ format. General information on how we cleaned our data is available in the online supplementary information.

### Data available in CIN

Table 2 outlines data items available in CIN between 2008 and 2018. Our table is based on the NPD user guide and data collection specifications as well as the NPD Data Tables, which contain a complete list of variables normally available across all NPD data collections (including the school census and CLA datasets).^10 13 16^ Further child-level descriptive statistics of the cohort, including breakdowns by census year and proportions of missing data, is provided in the online supplementary information. Note, in cases where children had multiple episodes over a given period, we have taken the first available episode information within the specified period.

**Table 2:** Variables available in CIN, between 2008/09 and 2017/2018

	Available information	2008/2009	2009/2010	2010/2011	2011/2012	2012/2013	2013/2014	2014/2015	2015/2016	2016/2017*	2017/2018*
Unique identifiers	Pupil matching reference/unique pupil number	✓	✓	✓	✓	✓	✓	✓	✓	✓	✓
	LA child ID	✓	✓	✓	✓	✓	✓	✓	✓	✓	✓
	LA ID†	✓	✓	✓	✓	✓	✓	✓	✓	✓	✓
Children’s characteristics	Age/date of birth/expected date of birth	✓	✓	✓	✓	✓	✓	✓	✓	✓	✓
	Ethnicity	✓	✓	✓	✓	✓	✓	✓	✓	✓	✓
	Gender	✓	✓	✓	✓	✓	✓	✓	✓	✓	✓
	Disability	✓	✓	✓	✓	✓	✓	✓	✓	✓	✓
	Date of death‡		✓	✓	✓	✓	✓	✓	✓	✓	✓
	Seeking asylum	✓	✓								
	Adopted from care	✓	✓								
	Income deprivation	✓									
Case information	Referral date	✓	✓	✓	✓	✓	✓	✓	✓	✓	✓
	Primary need status	✓	✓	✓	✓	✓	✓	✓	✓	✓	✓
	Case closure date§	✓	✓	✓	✓	✓	✓	✓	✓	✓	✓
	Reason for closure¶	✓	✓	✓	✓	✓	✓	✓	✓	✓	✓
	No further action after assessment		✓	✓	✓	✓	✓	✓	✓	✓	✓
	Date of initial child protection conference		✓	✓	✓	✓	✓	✓	✓	✓	✓
	Whether on child protection plan	✓									
	Referral source						✓	✓	✓	✓	✓
Child protection plan information	Child protection plan start date	✓				✓	✓	✓	✓	✓	✓
	Child protection plan end date	✓				✓	✓	✓	✓	✓	✓
	Category of abuse	✓				✓	✓	✓	✓	✓	✓
	No of previous child protection plans	✓				✓	✓	✓	✓	✓	✓
Service provision	Service type	✓									
	Service provider	✓									
	Service provision start date	✓									
	Service provision end date	✓									

*Data not released at the time of writing. Data availability is based on children in need census data collection specifications and existing data.

†There are some changes to LA IDs following changes to LA structure in 2008/2009 and 2009/2010.

‡Date of death is collected for children where services continue to be provided after a child has died.

§Case closure dates are not always available even when cases are closed. Instead case closure is indicated by ‘reason for closure’ or ‘no further action’.

¶‘No further action after core assessment’ was added as a reason for closure in 2009/2010.

LA, Local Authority.

### Unique identifiers

Child-level identifiers in the data include the PMR, which is derived from the unique pupil number (UPN) given to all children who enter the state school system in England (or in some cases earlier, for example, where a child receives an Education, Health and Care Plan for additional support (formerly a Statement of Special Educational Needs)). In their annual CIN returns to the Department for Education, LAs submit each child’s UPN where this has been assigned. As UPNs are classed as identifiable and sensitive information, the Department for Education anonymises UPNs to derive PMRs as child identifiers. Both CLA and school censuses include PMRs, meaning it is possible to link children with PMRs across all three datasets using a straightforward 1:1 deterministic linkage method. Children below school age (under age 5) will not usually have a PMR, nor will children exclusively educated outside the state sector (eg, in private schools or through homeschooling). In our data, 36% of children did not have a recorded PMR for these reasons.

Alternatively, children can be identified through LA child IDs which are unique identifiers used locally by LAs and are available for 100% of children in CIN. Because the same child IDs can be used by different LAs, unique IDs must be derived by combining child IDs with LA IDs. This can be used to link children in CIN to the CLA dataset. However, children acquire new LA child IDs when they move LAs, meaning these children can only be tracked longitudinally if they have PMRs. Children also acquire new LA child IDs and PMRs when they are adopted—meaning some children have multiple LA child IDs and PMRs which cannot be linked between census years. In 2015/2016, 5360 children were adopted from care,^9^ which is 0.9% of children in the 2015/2016 CIN.

Unique episodes (ie, referrals and open cases) can be identified with a combination of the referral date and the derived LA child ID.

### Children’s characteristics

CIN contains information on children’s gender, date of birth and ethnicity. Children in CIN through prebirth referrals are not attributed a gender, and children with intersex characteristics may be recorded as intersex. In our data (2008–2016), 50.2% of children were male, 47.5% female and around 2.3% unknown. A total of 2182 children were recorded as intersex or other. Sixty-six per cent of children in CIN are recorded as having a White ethnic background, followed by 7% Asian, 7% black and 6% mixed. The mean age at referral was 7.25 years, with a range of 0–25 years.

In terms of health-related information, CIN holds data on disability categories which are: autism/Asperger’s syndrome, behaviour, communication, consciousness, hand function, hearing, incontinence, learning, mobility, personal care, vision and any other. Further information on the coding criteria for disabilities is available under the Census Guide for LAs.^8^ Note, how LAs collect and code information on children’s disabilities have been known to vary, and validity/reliability issues are likely to present 17(see the Limitations section).

Geographical information at a level lower than LA is available for episodes between 2008 and 2010, but access to this information is severely restricted due to its sensitivity and risk of identification. Linkage to the lower layer super output area for children in the spring school census is possible but the justification for such information and permission from the Department for Education will be required (see the Data access section).

### Case information

Basic case information is available for all episodes, including date of referral, primary need status, case closure date and a reason for closure. Primary need status captures the main reason why a child has been referred for social care support. Information on referral source, including whether the referral came from health services, is available from 2013 to 2014 onwards.

Based on the first available referral information between 2008 and 2016, 45% of children were referred to children’s services due to suspected abuse or neglect, 17.8% due to family dysfunction and 9.8% due to family in acute stress. 4.8% of children were referred primarily due to their disabilities. Note that categorisation of primary need is hierarchical (eg, if there is suspected abuse or neglect, this is the category recorded irrespective of whether there are other reasons), represents the primary reason why a child was referred for/received support rather than their actual need, and only one reason can be recorded.^8^ Further information on the coding criteria for primary needs status is available under the Census Guide for LAs,^8^ and descriptive statistics for primary need status is available in the online supplementary information.

If children are judged to be under risk of significant harm, they may go onto child protection plans. For these groups of children, further information is recorded on a child protection plan start and end dates, and the reasons for the child protection plan (category of abuse). Between 2008 and 2016, 10.7% of children in our data went onto a child protection plan at some point. For a yearly breakdown of % children with child protection plans, see online supplementary information.

While ‘Date of Initial Child Protection Conference’ can be requested from the Department for Education, this information is missing for the majority of eligible children (see the Data quality section). Information on service provision, including service type and provider information, is available for 2008/2009 only.

### Data quality

Each year until 2011/2012, between one and three LAs did not submit data (eg, as a result of changes to their IT systems) meaning children in these LAs are missing from these years (the LAs affected are Leicestershire, Devon, Newham, Havering and the Isle of Wight; details are available in ‘Additional Issues by Year’ in the online supplementary information). Certain variables in our data extract had high levels of missing information: The ‘Date of Initial Child Protection Conference’ variable, available from 2009 to 2010, was missing for 92% of children known to have been placed on child protection plans. For episodes where we could infer case closure (see online supplementary information), around 18.5% of episodes between 2008 and 2016 were missing case closure dates. In contrast, children’s demographic information was relatively complete, with around 2% of gender and age details recorded as missing.

According to the Department for Education, children should only have one episode open at any one time.^18^ However, missing closure dates mean some consecutive episodes wrongly overlap, leading to multiple open episodes. Further, when children with open cases are rereferred, some LAs submit this information as a new referral. As episodes are only identifiable by referral dates, a single case can be wrongly recorded as multiple episodes with overlapping dates. Based on recorded and estimated closure dates in our data, we estimate that around 8.6% of episodes between 2008 and 2016 overlap with another episode.

The Department for Education states that their data quality has improved over time, with most recent records most likely to be correct.^18^ In our data, we found a reduction in our estimated percentage of missing case closure dates from 33% in 2008/2009 to 8% in 2015/2016. Our estimated percentages of overlapping episodes also decrease with time, from 23% in 2008/2009 to 7% in 2015/2016. Further details on missing data and errors are available in the online supplementary information.

### Findings to date

CIN data are used by the Department for Education for their national statistics on children in need, describing numbers and trends of children in need across England.^6^ In their latest statistical release, there were 389 430 children ‘in need’ and 51 080 children on child protection plans on 31 March 2017. 12.9% of children in need on the 31 March 2017 had disabilities recorded.^6^ In a different part of government, the Department for Communities and Local Government (DCLG) has used CIN, together with several administrative datasets, to evaluate the Troubled Families Programme.^19^ Introduced in 2012, the Troubled Families Programme is an England-wide intervention scheme targeting ‘troubled families’ with repeated/multiple presentations to local government services across social care, health and police. DCLG used CIN to track families going through the programme between 2012 and 2015, and to select a comparable sample of families who did not go through the programme. Overall, it found that the programme had minimal impact on family outcomes.^19^

Away from the government, researchers have used CIN with linkage to other data sources. In one study, CIN data were linked to the Index of Multiple Deprivation (a small-area measure of material deprivation), finding a clear association between higher overall levels of deprivation and higher rates of intervention at LA level.^20^ However, at neighbourhood level, families living in LAs with lower overall levels of deprivation were more likely to experience children’s service interventions compared with families living in LAs with higher overall levels of deprivation.^20^ Researchers have used CIN together with CLA and school censuses, finding that the educational outcomes of children in need are generally significantly poorer than the general population and in fact not dissimilar to children in care.^21^

## Strengths and limitations

### Strengths

CIN is the most complete data source of potentially vulnerable children in England. CIN holds information on all children referred to LAs and consequently accepted for a needs assessment, not just children assessed as in need.^18^ This means CIN holds potential for capturing information on children who are at the ‘edge of social services:’ children over the threshold to be ‘accepted’ for an assessment, but under the threshold of need for statutory LA support and intervention. CIN also holds several data items on disabilities, including children who are primarily referred to children’s services due to parental disabilities (but see the Limitations section).

As a longitudinal dataset, CIN can be used to track such children through time. Linkage to the school census and CLA is likely to capture a near-complete pathway of children through education and social care services, as well as providing limited information on their outcomes such as school attendance and educational outcomes.^11 12^ A recent scoping report by authors (MAJ and JW) also highlights the linkage potential with a range of existing administrative datasets from health, family justice, tax records and criminal justice, as well as additional LA data.^12^ With participant consent, researchers may be able to link CIN to their own dataset if they hold children’s UPNs (school IDs) or LA child IDs. CIN also holds information on children’s date of births which may be useful for data linkage, but note that access to this information is severely restricted.

Currently, researchers are using CIN, together with the school census, Hospital Episode Statistics and death registration records, to evaluate a home-visiting programme on child maltreatment.^22^ Authors are using the linked CIN-CLA-School Census datasets to examine variation in children’s educational outcomes (MAJ), and investigate variations in referral rates from health services to children’s services in England using CIN (EHE and JW).^23^ At the time of writing, there are several ongoing studies using CIN.

### Limitations

As an administrative dataset, there is relatively little metadata available for CIN. While authors hope this paper will address some of this limitation, it can be challenging to understand the structure of CIN and the nature of the available information without knowledge of children’s social care systems in England.

The PMR, which is a nationally unique to individual children, is only available for children who have entered state school (or in some circumstances earlier) and is therefore not available for a significant proportion of CIN (36% in our dataset). Unique identification of all children is cross-sectionally possible through the LA child ID. However, if a child moves between LAs, they will be assigned a new local ID, and therefore, that child’s records will not link longitudinally. In addition, all new identifiers are assigned when a child is adopted meaning that records postadoption cannot be linked longitudinally with records preadoption.

CIN does not contain information on the type nor intensity of services provided to children. An open case does not necessarily mean a child is actively being supported by LAs, meaning it is difficult to distinguish the level of need and support within CIN. While CIN includes children who are accepted for needs assessments and consequently assessed as not in need, it does not include children receiving ‘early help’ support. This means CIN does not capture a full picture of those ‘on the edge of social services.’

CIN lacks family information such as socioeconomic details and parental characteristics.^20^ (Note, information on free school meals and area-based socioeconomic indicators is available through the school census if children have PMRs.) Information on relatedness is also unavailable, meaning siblings and relatives cannot be identified within the dataset.

Finally, the Department for Education has noted variations in recording practices between LAs, as well as between census years.^18^ This means data are not necessarily comparable between LAs and census years. For example, some LAs conduct two assessments of children to assess their needs, while others conduct one continuous assessments (see online supplementary information). Similarly, we are aware of variations in referral and assessment practices between LAs (which are not properly documented or understood at present), and the threshold of entry into the CIN data is likely to vary between areas. Variables which require decision-making around coding, such as disability categories^24 25^ and primary needs statuses,^8^ may be particularly susceptible to validity and reliability issues within and between areas. For example, different incentives surround the primary need status categories, in that child protection cases with a risk of significant harm (likely associated with abuse and/or neglect) are prioritised for children’s services assessment and support. This means the ‘abuse or neglect’ category could be selected by front-line staff as a bid to prioritise cases.

Given these limitations, researchers should be cautious around the interpretation of CIN and any inferences that are drawn from its analyses. To address these limitations, further research is required to better understand the meaning of CIN data—in other words, what exactly are the variables capturing?

## Data access

Adverse childhood experiences are detrimental for health and development, but prospective research with potentially vulnerable children is challenging due to practical and ethical issues.^1–4^ CIN is an ongoing, longitudinal data source which provides researchers with an opportunity to access data on such potentially vulnerable children in England.

Access to CIN extracts can be requested through the Department for Education NPD team. Data from the most recent census period typically become available for researchers in the following Spring, after the official statistics on the characteristics of CIN is updated by the Department for Education.^6^

The application process and guidelines, which have changed in 2018/2019, are outlined online at (https://www.gov.uk/guidance/how-to-access-department-for-education-dfe-data-extracts). At present, the Department for Education supplies CIN extracts to any person or organisation whose aim is to promote the education or well-being of children in England by (1) conducting research or analysis, (2) producing statistics and/or (3) providing information, advice or guidance.^10^ Applicants will be asked to outline how their project serves the public benefit, select the legal basis for data sharing as well as the conditions for processing data under the General Data Protection Regulation. They will also be asked to specify each individual variable requested using a standard template provided by the Department for Education and access to particularly sensitive data items will require justification. Due to the vulnerabilities associated with children in need, the CIN dataset as an entirety is considered ‘tier 1’, attributed to the most sensitive personal information. The Department for Education has stated that access to data will in most cases be through the Office for National Statistics (ONS) Secure Research Service. Researchers using these resources will require ONS approval as well as a certificate from the Disclosure and Barring Service (ie, background check of police records). Depending on the data requested, it may take several months for the data request to be approved. Researchers must also comply with relevant data protection law, and must store the data within the European Union.

These stringent data protection policies and practices are in place to ensure the rights and identities of the data subjects (ie, children referred to LAs) are protected. As the data are individual level, once access has been granted, researchers have further ethical and legal obligations to maintain the confidentiality of the data and ensure that disclosure risk is minimised. This includes complying with Department for Education rules on the suppression of small cell counts or any other information that might result in secondary identification of a data subject.
