# Supplementary material for: Cohort profile: Children in Need Census (CIN) records of children referred for social care support in England
Source: BMJ Open. 2019 Feb 22;9(2):e023771. doi: 10.1136/bmjopen-2018-023771 (PMC6398790; doi:10.1136/bmjopen-2018-023771)
Supplement: Supplementary data [file bmjopen-2018-023771supp001.pdf]

Data Profile: Children in Need Census (CIN) Supplementary Information

Authors: Emily H. Emmott*[1], Matthew A. Jay[2,3], Jenny Woodman[1]

Affiliation: [1]Thomas Coram Research Unit, Department of Social Science, UCL Institute of Education, University College London, UK [2]UCL Great Ormond Street Institute of Child Health, University College London, UK, [3]Department of Anaesthesia and Pain Medicine, Great Ormond Street Hospital for Children NHS Foundation Trust, London, UK

Corresponding Author Contact*: emily.emmott@ucl.ac.uk

Contents

About our data 2
General Information on or Data Cleaning Method 2
LA Child ID 2
Pupil Matching Reference 2
Descriptive Statistics by Year 4
Children's characteristics 4
Case Information 5
Missing case closure dates 6
Known Data Issues 7
General Issues 7
Additional Issues by Year 8
References 8

About our data

In November 2017, we received complete episode information available in CIN at the time of our request from DfE. This amounted to information on all cases submitted by LAs up to and including the 2015/16 census. In addition, we requested and received information on children's demographic characteristics, primary need status, child protection plan start and end dates, and referral source.

General Information on our Data Cleaning Method

Here we provide a verbal description of our data cleaning method. For further information, please contact the corresponding author.

LA Child ID

Some children in the CIN data share Child IDs, where the same Child ID is used by two or more LAs. To derive LA-specific unique child IDs, new LA Child IDs were created by:

1. Creating a consistent LA ID across all census years. In 2009/10, Bedfordshire (LA ID 820) split into Bedford (LA ID 822) and Central Bedfordshire (LA IF 823), and Cheshire (LA ID 975) split into Cheshire East (895) and Cheshire West & Chester (896). For consistency, Bedford and Central Bedfordshire was reclassified as Bedfordshire, and Cheshire East and Cheshire West & Chester was reclassified as Cheshire.
2. Creating an LA-specific child ID: The original Child ID was merged with the new, consistent LA ID as outlined in point 1.

Overall, this led to an increase from 1,995,672 Child IDs in the dataset to 27,611,54 LA Child IDs in the dataset (increase of an estimated 765,482 children, or 38%).

Pupil Matching Reference

Some children in the CIN data have missing PMRs, while others have multiple PMRs. These issues, where possible, were addressed by:

1. Filling the "missing PMRs": For each LA Child ID, we identified cases where PMRs were recorded in at least one episode but also missing in at least one episode. We then replaced missing records with the available PMR.
2. Replacing "different PMRs": For each LA Child ID, we identified cases where children had more than one PMR attributed. We replaced all PMR records with the first recorded PMR in the dataset.

Overall, this led to a reduction in **the number of PMRs** from 1,808,417 to 1,761,224 (reduction of 47,193 PMRs; 2.6%) and a reduction in the **number of missing PMR records** from 1,841,603 to 1,474,797 (reduction of 366,806 rows; 20%).

Child's Age at Referral

We received information on children's month and year of birth. Child's age at referral was estimated from the referral date and month/year of birth. Some children in CIN had impossible birth dates, particularly for those in the first census submission. Impossible ages at the time of referral were recoded as missing. We derived children's age at referral by:

1. Recoding cases with referral dates earlier than 2nd April 1983 as missing, as these cases are likely to be errors (increase of missing in 606 rows; 0.01%).
2. We only had the month and year of birth for each child. We therefore set the 15th of each month to estimate the child's birth day. We used this date and the referral date to calculate their age at referral (in months and years).
3. When age at referral was more than 7 months before birth (-7 months old), we recoded this a missing as it is unlikely for pre-birth referrals to occur in the first trimester. Children who were referred between 6 to 0 months before birth (-6 to 0 months old) were revalued as being referred at age 0. We also revalued referrals over 26 years as missing, as this is most likely to be an error (increase of missing in 15434 rows; 0.2%).

Dates

The Department of Education's CIN submission tool flags missing dates, meaning LAs may "fill" missing dates with unlikely values. Some dates are impossible, and these were recoded as missing:

1. Child Protection Plan Start Dates of 1st January 1900 was recoded as missing (increase of missing in 1 row).
2. Child Protection Plan End Dates of 1st January 1900 was recoded as missing (increase of missing in 50468 rows; 0.8%)
3. Referral Dates of 5th February 7943 was recoded as missing (increase of missing in 1 row).

Primary Need Status (Primary Need Code)

There were minor coding errors in Primary Need Status. We recoded the Primary Need Status into the following:

- n1 = N1
- A2, n2 = N2
- A3, n3 = N3
- A4, n4 = N4
- A5, n5 = N5
- n6 = N6
- n7 = N7
- n8 = N8
- N0, NO, UnmappedNeedCode, UNMAPPEDNEEDCODE, NA = N0

Ethnicity

There were minor coding errors in Ethnicity. We recoded Ethnicity into the following:

- NA, UNCL, MISS, N/A, INVA = 'Missing'
- AOEG = 'Other'

Descriptive Statistics by Year

The following tables present basic descriptive statistics from our child-level CIN data, by year. Our CIN data contains all episodes submitted by LAs, up to 31st March 2016. We supress small values in each cell with *, in line with the Department for Education's CIN data usage conditions. In cases where children had multiple episodes, **we have taken the earliest episode information within the specified time period**.

Children's characteristics

Census Year	08/09	09/10	10/11	11/12	12/13	13/14	14/15	15/16	Other	Total
N Child	489501	659146	651676	852205	859518	899799	904613	886518	2895	2761155
Prop. Total Children (by census year)	0.18	0.24	0.24	0.31	0.31	0.33	0.33	0.32	0.00	1
**Gender**	N *(%)*	N *(%)*	N *(%)*	N *(%)*	N *(%)*	N *(%)*	N *(%)*	N *(%)*	N *(%)*	N *(%)*
Male	261543 *(53)*	347410 *(53)*	342352 *(53)*	439332 *(52)*	444383 *(52)*	463367 *(51)*	465229 *(51)*	453349 *(51)*	1602 *(55)*	1385500 *(50)*
Female	223404 *(46)*	310550 *(47)*	307217 *(47)*	399967 *(47)*	404077 *(47)*	424421 *(47)*	426991 *(47)*	416181 *(47)*	1292 *(45)*	1310379 *(47)*
Unknown	4274 *(1)*	1082 *(0)*	2035 *(0)*	12638 *(1)*	10809 *(1)*	11707 *(1)*	11778 *(1)*	16254 *(2)*	0 *(0)*	63092 *(2)*
Intersex/Other	279 *(0)*	103 *(0)*	71 *(0)*	267 *(0)*	248 *(0)*	303 *(0)*	614 *(0)*	732 *(0)*	0 *(0)*	2182 *(0)*
**Referral Age**										
Mean	7.768	8.72	8.65	7.60	7.55	7.62	7.72	7.96	7.95	7.73
Median	7.42	8.67	8.58	7.08	7.00	7.08	7.25	7.58	7.83	7.25
Min (in years)	0	0	0	0	0	0	0	0	0	0
Max (in years)	25	24	25	25	25	25	25	25	19	25
Lower Quartile	3.17	4.75	4.58	2.83	2.83	2.92	3.08	3.33	3.33	3.00
Upper Quartile	12.33	13.00	13.00	12.25	12.17	12.25	12.33	12.50	12.42	12.42
N Missing	5829	1905	2236	9139	9104	9643	9970	14653	20	59469

**Ethnicity**	N *(%)*	N *(%)*	N *(%)*	N *(%)*	N *(%)*	N *(%)*	N *(%)*	N *(%)*	N *(%)*	N *(%)*
Asian	28664 *(5.9)*	38961 *(5.9)*	38432 *(5.9)*	51636 *(6.1)*	53009 *(6.2)*	58086 *(6.5)*	61645 *(6.8)*	62964 *(7.1)*	141 *(4.9)*	194268 *(7.0)*
Black	34964 *(7.1)*	44862 *(6.8)*	42823 *(6.6)*	57554 *(6.8)*	59736 *(6.9)*	63559 *(7.1)*	68204 *(7.5)*	68820 *(7.8)*	191 *(6.6)*	198717 *(7.2)*
Chinese	6499 *(0.2)*	1223 *(0.2)*	1215 *(0.2)*	1681 *(0.2)*	1600 *(0.2)*	1745 *(0.2)*	1769 *(0.2)*	1794 *(0.2)*	*	*
Mixed	30416 *(6.2)*	37378 *(5.7)*	39293 *(6.0)*	53300 *(6.3)*	55287 *(6.4)*	66518 *(6.5)*	61895 *(6.8)*	61853 *(7.0)*	257 *(8.9)*	152777 *(5.5)*
Other	11123 *(2.3)*	13025 *(2.0)*	12031 *(1.8)*	16767 *(2.0)*	16267 *(1.9)*	17251 *(1.9)*	18612 *(2.1)*	19391 *(2.2)*	*	*
White	352140 *(71.9)*	475575 *(72.2)*	480390 *(73.7)*	603626 *(70.8)*	610220 *(71.0)*	634026 *(70.5)*	630921 *(69.7)*	616809 *(69.6)*	2141 *(74.0)*	1821193 *(66.0)*
Missing	31162 *(6.4)*	48121 *(7.3)*	37491 *(5.8)*	67640 *(7.9)*	63398 *(7.4)*	66518 *(7.4)*	61566 *(6.8)*	54886 *(6.2)*	111 *(3.8)*	325806 *(11.8)*

Case Information

**Census Year**	**08/09**	**09/10**	**10/11**	**11/12**	**12/13**	**13/14**	**14/15**	**15/16**	**Other**	**Total**
**N Child**	489501	659146	651676	852205	859518	899799	904613	886518	2895	2761155
**Prop Total Children in CIN, 08/16**	0.18	0.24	0.24	0.31	0.31	0.33	0.33	0.32	0	1
**Primary Need Status**	N *(%)*	N *(%)*	N *(%)*	N *(%)*	N *(%)*	N *(%)*	N *(%)*	N *(%)*	N *(%)*	N *(%)*
N0 (not stated)	64783 *(13.2)*	98309 *(14.9)*	68814 *(10.6)*	77568 *(9.1)*	70184 *(8.2)*	77257 *(8.6)*	78370 *(8.7)*	72596 *(8.2)*	1278 *(44.2)*	391639 *(14.2)*
N1 (Abuse or neglect)	184005 *(37.6)*	252801 *(38.4)*	277143 *(42.5)*	383437 *(45.0)*	402492 *(46.8)*	425509 *(47.3)*	446578 *(49.4)*	457511 *(51.6)*	897 *(31.0)*	1242452 *(45.0)*
N2 (Child disability / illness)	55611 *(11.4)*	61497 *(9.3)*	61281 *(9.4)*	63845 *(7.5)*	62001 *(7.2)*	58272 *(6.5)*	56755 *(6.3)*	52471 *(5.9)*	265 *(9.2)*	131445 *(4.8)*
N3 (Parental disability / illness)	16894 *(3.5)*	19726 *(3.0)*	18577 *(3.0)*	24818 *(2.9)*	23758 *(2.8)*	23577 *(2.6)*	23169 *(2.6)*	22719 *(2.6)*	55 *(1.9)*	69997 *(2.5)*
N4 Family in acute stress	50410 *(10.3)*	69982 *(10.6)*	66963 *(10.3)*	86060 *(10.1)*	88511 *(10.3)*	88369 *(9.8)*	82239 *(9.1)*	74395 *(8.4)*	128 *(4.4)*	269954 *(9.8)*

N5 Family dysfunction	77580<br>*(15.8)*	110989<br>*(16.8)*	113522<br>*(17.4)*	162582<br>*(19.1)*	163198<br>*(19.0)*	175839<br>*(19.5)*	168028<br>*(18.6)*	158107<br>*(17.8)*	146<br>*(5.0)*	492231<br>*(17.8)*
N6 Socially unacceptable behaviour	10839<br>*(2.2)*	15536<br>*(2.4)*	15615<br>*(2.4)*	18377<br>*(2.2)*	16852<br>*(2.0)*	19106<br>*(2.1)*	19192<br>*(2.1)*	19036<br>*(2.1)*	46<br>*(1.6)*	60204<br>*(2.2)*
N7 (Low income)	3362<br>*(0.7)*	3836<br>*(0.6)*	3373<br>*(0.5)*	4299<br>*(0.5)*	4255<br>*(0.5)*	4509<br>*(0.5)*	4229<br>*(0.5)*	3789<br>*(0.4)*	*	*
N8 (Absent parenting)	17259<br>*(3.5)*	17039<br>*(2.6)*	17097<br>*(2.6)*	19962<br>*(2.3)*	17958<br>*(2.1)*	16629<br>*(1.8)*	16320<br>*(1.8)*	17361<br>*(2.0)*	46<br>*(1.6)*	50335<br>*(1.8)*
N9 (Cases other than CIN)	8758<br>*(1.8)*	9430<br>*(1.4)*	8290<br>*(1.3)*	11256<br>*(1.3)*	10308<br>*(1.2)*	10731<br>*(1.2)*	9732<br>*(1.1)*	8532<br>*(1.0)*	*	*
**Ever on a Child Protection Plan**	N<br>*(%)*	N<br>*(%)*	N<br>*(%)*	N<br>*(%)*	N<br>*(%)*	N<br>*(%)*	N<br>*(%)*	N<br>*(%)*	N<br>*(%)*	N<br>*(%)*
Yes	57530<br>*(11.8)*	67014<br>*(10.2)*	78292<br>*(12.0)*	125435<br>*(14.7)*	163184<br>*(19.0)*	189060<br>*(21.0)*	201253<br>*(22.2)*	190064<br>*(21.4)*	323<br>*(11.2)*	294153<br>*(10.7)*
No	431971<br>*(88.2)*	592131<br>*(89.8)*	573383<br>*(88.0)*	726769<br>*(85.3)*	696333<br>*(81.0)*	710738<br>*(79.0)*	703359<br>*(77.8)*	696453<br>*(78.6)*	2571<br>*(88.8)*	2467002<br>*(89.3)*

Missing case closure dates

While some episodes have no case closure information, meaning identifying missing case closure dates can be challenging, we can infer case closure from various characteristics of the record. We assumed case closure if:

1. An episode (identified through LA Child ID and referral date) had any record of case closure (case closure date, no further action record, or reason for closure record).
2. If an episode ceased to be included in following census years, indicating that the episode was no longer active.

The following table outlines the descriptive for missing case closure dates, for cases we identified as having closed.

**Census year**	**08/09**	**09/10**	**10/11**	**11/12**	**12/13**	**13/14**	**14/15**	**15/16**	**Other**	**All Years**
**N Episodes**	563,857	794,798	758,002	1,001,578	1,001,650	1,049,397	1,047,014	1,001,221	2,905	4,668,623
**N missing case closure date**	101,510	114,903	77,201	106,116	100,324	91,977	86,186	467,68	138,365	863,350
**% missing case closure dates (by census year)**	33%	26%	18%	17%	16%	14%	13%	8%	46%	18%

Known Data Issues

General Issues

1. **Non-eligible cases:** There are a small number of episodes which should not be included in the dataset according to the data collection specification, where the case closure date falls before 1st April 2008. We have identified 1076 such cases (<0.1%).

2. **Duplicate records:** Children should have only one open episode ant any one time. However, some LAs report duplicate episodes due to their reporting systems (for example, a new referral of a child already on the CIN database may incorrectly lead to a new episode opening). DfE estimates around 0.07% of episodes are duplicated(1).

3. **Data quality:** The number of child protection plans on the census date at any given year does not equate to (number of child protection plans on the previous census date) + (the number child protection plans starting within the year) – (number of child protection plans ending within the year). DfE states data errors are likely due to improving data quality and reporting year on year, with assumption that the most recent data is the most accurate(1).

4. **Recording variation:**
    a. There is significant variation between LAs on referrals which have no further action. DfE notes that this could be because of practice differences between LAs, where some LAs do not accept referrals for assessment under a certain threshold (and consequently is not included in CIN), while others record all referrals received(1).
    b. From 2011/12, some LAs have been involved in trials around new approaches to assessments, where they do not distinguish between initial and core assessments. Table S1 outlines the LAs involved in the trials by year, up to 2016/17. For these LAs, the recording of "no further action after assessment" may vary compared to other LAs.

Table S1: LAs involved in assessment trial by year.

LA	2011/12	2012/13	2013/14	2014/15	2015/16	2016/17
Cumbria	✓	✓	Opted out end of April 2013			
Hackney	✓	✓	✓	✓	✓	✓
Hammersmith and Fulham	✓	✓	✓	✓	✓	✓
Islington	✓	✓	Opted out end of April 2013			
Kensington and Chelsea	✓	✓	✓	✓	✓	✓
Knowsley	✓	✓	✓	✓	✓	✓
Wandsworth	✓	✓	✓	✓	✓	✓
Westminster	✓	✓	✓	✓	✓	✓
Hartlepool			✓ (Opted in Oct 2013)	✓	✓	✓

Additional Issues by Year

2008/09

- Leicestershire and Devon did not submit data for this census year.

2009/10

- Leicestershire did not submit data for this census year.

2010/11

- Newham, Havering and Isle of Wight did not submit data for this census year.

2011/12

- Newham and Havering did not submit data for this census year.

2016/17

- Ealing reported issues with their information on child protection plan reviews. Consequently, DfE excluded Ealing from their annual statistics on the number and percentage of child protection plans that were reviewed(1).
- Slough changed their referral recording system. Up to 2015/16, Slough only recorded referrals which led to an assessment. From 2016/17, Slough recorded any referral/contact, irrespective of whether the child was assessed(1).

References

1. Department for Education. Characteristics of children in need in England: 2016 to 2017. Data Quality and Uses. 2017.
